# Hierarchical Structure of iPP During Injection Molding Process with Fast Mold Temperature Evolution

**DOI:** 10.3390/ma12030424

**Published:** 2019-01-30

**Authors:** Vito Speranza, Sara Liparoti, Roberto Pantani, Giuseppe Titomanlio

**Affiliations:** Department of Industrial Engineering, University of Salerno–via Giovanni Paolo II, 132, 84084 Fisciano (SA), Italy; vsperanza@unisa.it (V.S.); rpantani@unisa.it (R.P.); gtitomanlio@unisa.it (G.T.)

**Keywords:** morphology, injection molding, cylindrites, mold temperature

## Abstract

Mold surface temperature strongly influences the molecular orientation and morphology developed in injection molded samples. In this work, an isotactic polypropylene was injected into a rectangular mold, in which the cavity surface temperature was properly modulated during the process by an electrical heating device. The induced thermo-mechanical histories strongly influenced the morphology developed in the injection molded parts. Polarized optical microscope and atomic force microscope were adopted for morphological investigations. The combination of flow field and cooling rate experienced by the polymer determined the hierarchical structure. Under strong flow fields and high temperatures, a tightly packed structure, called shish-kebab, aligned along the flow direction, was observed. Under weak flow fields, the formation of β-phase, as cylindrites form, was observed. The formation of each morphological structure was analyzed and discussed on the bases of the flow and temperature fields, experienced by the polymer during each stage of the injection molding process.

## 1. Introduction

Injection molding is a widespread process for the mass transformation of polymeric materials into final usable objects. During such a process, the polymer is melted and injected into a cavity with the shape of the desired objects, where additional material is fed into the cavity to compensate for the shrinkage due to the solidification. After that, cooling up to the extraction temperature takes place [1]. During the injection molding stages, the polymer chains are subjected to intensive shear and elongational flow fields that, on their turn, influence the solidification process and the morphology developed both along the flow and transverse directions [2]. The shear experienced by the polymer is not homogeneous along the cavity thickness; in particular, it is known that the shear is larger at the cavity wall than in the inner parts of the cavity. The shear and the molecular stretch undergone by the polymer chains determine, together with the cooling rate, the morphology developed in a certain area of the molded objects. Under high shear stresses, anisotropic shish-kebab morphology is observed, whereas in quiescent conditions, isotropic spherulitic morphology is found [3,4,5]. Different morphological structures give rise to a distribution of mechanical properties. Thus, understanding the mechanism that regulates the formation of any morphology with the aim of modulating mechanical properties of the polymeric objects is of great interest [6,7,8,9,10]. 

Fujiyama et al [11] proposed a model for the formation of the shish-kebab morphology. When a flow field is applied, coiled polymer chains undergo an extension, attaining a high degree of alignment that induces the crystallization into shish. The chains that do not reach a high degree of alignment, the kebabs, crystallize epitaxially on the shish, giving rise to chain–folded structures.

Pogodina et al. observed that structures aligned with the flow, the shish, appear in isotactic polypropylene (iPP) already at 148 °C, applying a step shear of 10 s^−1^ [12]. Both the molecular orientation in the melt, and the crystallization degree in the solid increase with shear rate and shear duration. Short duration high shear rate is more effective in orienting molecules than long duration small shear rate.

Jeu et al suggested that under flow the formation of shish precursors occurs, namely a mesophase, promoting the nucleation process, which induces the successive formation of shish [13]. 

All these works suggested that when the polymer chains undergo a huge flow field, they crystallize in the form of shish; namely, structures aligned along the flow direction, at temperatures significantly higher than those observed in quiescent conditions. Even if many works were carried out to analyze the mechanism that induces the formation of different morphological structures [4,14,15], the conditions that allow their formation during industrial polymer processes are still under debate [16].

In this paper, a deep characterization of morphology has been carried out on injection molded samples. The samples have been obtained coupling the injection molding with a system that allows modulating the cavity surface temperature during the process. Such a system causes, due to joule effect, the increase of cavity surface temperature up to the selected values within a few seconds. Different cavity surface temperatures and heating times have been adopted during the production of the molded samples, with the aim of analyzing the effects of the temperature, and the flow fields, on the morphology developed along the sample thickness. Also, mechanisms that allow the formation of shish-kebab morphology have been proposed, and related with the flow and temperature fields undergone by polymer chains during the process. 

The formation of different morphological structures is also related to the formation of different crystalline phases when the polymer chains experience certain temperature and flow fields. There are three types of crystal phases found in iPP; namely α, β, and γ-phase [17]. γ-phase can be found only when pressures higher than 2000 bar are achieved [18]. α-phase is characteristic of most of the parts of the molded samples, since it is the most stable one, whereas β-phase is thermodynamically metastable, and more difficult to be found [19]. The high shear stresses experienced by the polymer chains during the injection molding process mainly induces the transformation of β-phase into α-phase [20]. However, the β-phase formation is highly desired, since it enhances the toughness of the molded samples. Generally, this increase of toughness has been achieved by adding β-nucleating agents during the process [21]. 

In this paper, the possibility to drive the formation of different crystalline phases, with higher attention to the formation of β-phase, has been discussed on the bases of the adopted operating conditions.

## 2. Materials and Methods 

The T30G polypropylene (iPP) commercial grade (Basell, Ferrara, Italy) was adopted for the injection molding tests. This material was characterized concerning rheology, and crystallization in previous works [22,23,24,25]. 

**Figure 1:**
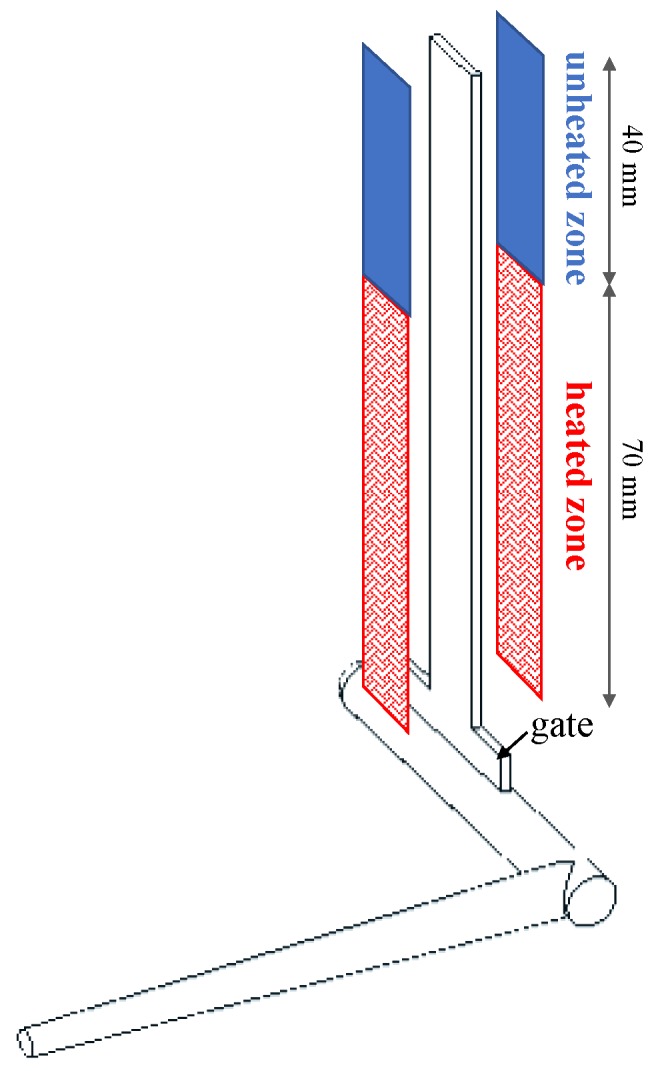
Sketch of the cavity adopted for the injection molding tests, with the heating device.

Figure 1 schematizes the polymer flow path during the process. The path includes the sprue, the runner, the gate and the cavity. The first 70 mm of the cavity, downstream the gate, undergo temperature cycles due to the presence of a heating device located just below the cavity surface. The heating device, characterized elsewhere [26,27], is mainly composed of a conductive layer (80 µm thickness) that induces the increase of temperature up to values (T_cs_) intermediate between the mold temperature and the injection temperature; the electrically conductive layer is sandwiched by two insulating layers (140 µm thickness on the mold side, and 20 µm thickness on the cavity side). A steel layer (100 µm thickness) protects the heating device from the incoming melt. The cavity dimensions are 110 mm length, 12.7 mm width, and 1.50 mm thickness, as shown in Figure 1.

The injection molding tests were performed with 220 °C melt injection temperature, 2.9 cm^3^ s^−1^ average volumetric flow rate (the cavity filling time was about 0.7 s), and 25 °C mold temperature. 720 bars were adopted during the holding stage. Table 1 summarizes all the operating conditions. The tests named Passive were performed without activating the heater, the Steel tests were performed replacing the heating device with a steel layer of the same thickness. In Table 1, the name of each test is composed by the temperature T_cs_ in °C measured on the cavity surface after 6 s heating time (T_cs_ = 80 °C and 150 °C for the tests considered in this work), and the heating time t_h_ in seconds. Examples of the temperature evolutions acquired during tests with 4 W/cm^2^ are shown in Figure S1 of the Supplementary Materials.

The activation time, *t_a_*, and the heating time, *t_h_*, represent the time during which the heating device was kept active before the first contact of the melt with the cavity surface, and the time during which the heating device was active after the contact of melt with the cavity surface, respectively.

The comparison reported in this work concerning the morphology developed along the sample thickness refers to the position at 15 mm downstream the cavity entrance.

The samples were cut along the flow direction, parallel to the flow-thickness plane, and chemically etched, following a procedure reported elsewhere [28]. Atomic Force Microscope (AFM) investigations were conducted in air and at room temperature with a Bruker Dimension instrument (Multimode Dimension V, Veeco, Santa Barbara, CA, USA) coupled with a Nanoscope V controller operating in tapping mode. Commercial probe tips with nominal spring constants of 42 N m^−1^, resonance frequencies of 300 kHz, and tip radius of 7 nm were used.

Sample slices were also analyzed by optical microscopy in polarized light by an Olympus BX51 microscope (OM, Olympus Italia S.R.L., Segrate, Italy) with crossed polarizer-analyzer. Optical micrographs were taken with sample slices oriented at 45° with respect to the analyzer.

The simulation software for the injection molding process was developed at University of Salerno (Fisciano, SA, Italy) [24], adapted to evaluate the flow rate during the injection molding process.

## 3. Results

**Figure 2:**
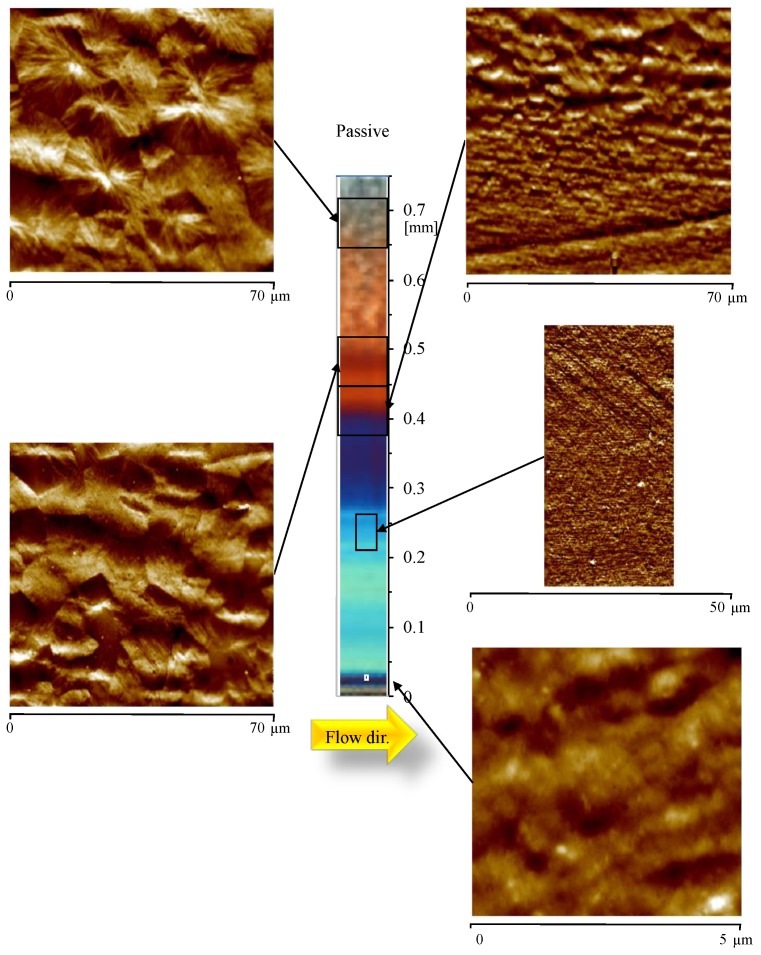
Optical micrograph and AFM height maps, at different distances from the sample surface, of the sample Passive, at 15 mm downstream the gate.

Figure 2 shows the morphology developed during the injection molding process, with the heating device adapted as an insulating layer, namely for the Passive test. The polarized optical micrograph (OM) and the AFM topographic maps for each position along the sample thickness are shown in the same figure. For each AFM map, the distance from the sample surface is reported. The OM displays a distinct skin-core morphology, characteristic of the injection molding process. The AFM maps allow obtaining more detailed information about the structure developed along the sample thickness. Within a small distance from the sample surface, 0.03 mm, a poorly organized structure was detected. From 0.03 mm to 0.24 mm from the sample surface, AFM shows a highly-oriented layer composed of structures aligned along the flow direction, having an average thickness in the range 100–300 nm. The layer that appears brown in the OM, between 0.40 mm and 0.47 mm, is composed of thick (more than 300 nm thickness) aligned structures characterized by significant lateral growth. The core is composed of randomly dispersed spherulites, as clearly visible in the AFM map at 0.67 mm, with a mean diameter of 20 ± 5 µm. 

**Figure 3:**
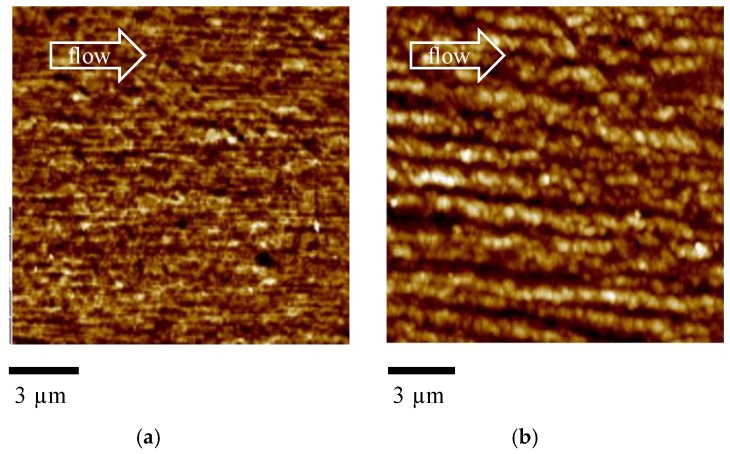
Enlargement of the AFM height map at (**a**) 0.24 mm and (**b**) 0.4 mm distance from the sample surface for the Passive sample.

Figure 3 shows a magnification of the oriented zone at 0.24 mm and at 0.40 mm from the sample surface.

Figure 3a mainly shows tightly packed structures aligned along the flow direction. Figure 3b also shows thick structures aligned along the flow direction, with average thickness ranging between 300 and 1000 nm. Comparison between the two figures clearly confirms that the thickness of the structures aligned along the flow direction increases with the distance from the sample surface.

**Figure 4:**
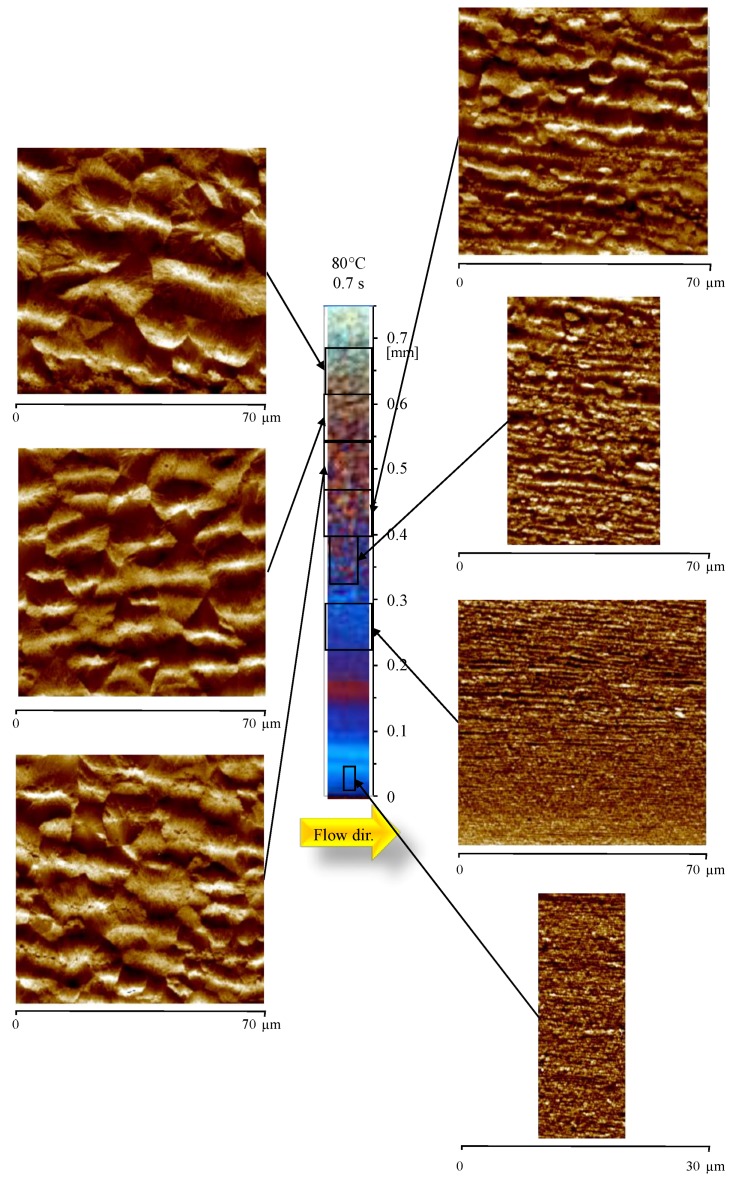
Optical micrograph and AFM height maps, at different distances from the sample surface, of the sample 80-07, at 15 mm downstream the gate.

Figure 4 shows the OM and the AFM height maps of the sample 80-07, obtained adopting 80 °C as cavity surface temperature during the cavity filling (0.7 s). 

**Figure 5:**
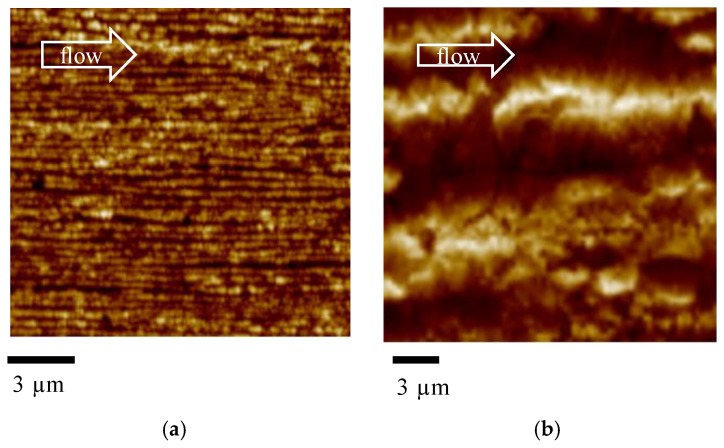
Magnification of the AFM height map at (**a**) 0.25 mm and (**b**) 0.50 distance from the sample surface for 80-07 sample.

The OM displays, again, a skin-core morphology; however, the width of colored bands is different with respect to the Passive sample, shown in Figure 2. This is mainly due to the different distribution of the orientation levels. Adopting high temperatures on the cavity surface during the filling, structures oriented along the flow direction were detected already at the sample surface. The AFM map at 0.25 mm shows that thin (smaller than 300 nm thickness) structures aligned along the flow direction can be observed up to 0.30 mm from the sample surface. Again, the thickness of these structures increases as the distance from the sample surface increases. Comparison of OM and AFM images shows that the structures aligned along the flow direction are also detectable in the layers where OM seems to show spherulitic structures. The AFM map at 0.50 mm from the sample surface shows thick (up to 6 µm thickness) structures aligned along the flow direction, characterized by significant lateral growth in the transverse direction (orthogonal to the flow front). The aligned structures disappear at 0.57 mm from the surface, where only randomly distributed spherulites can be observed. A magnification of the oriented layers at 0.25 mm, and at 0.50 mm is shown in Figure 5a,b, respectively.

As already observed for the Passive case, the larger the distance from the sample surface, the larger the lateral growth (namely, the thickness of the aligned structures increases). 

**Figure 6:**
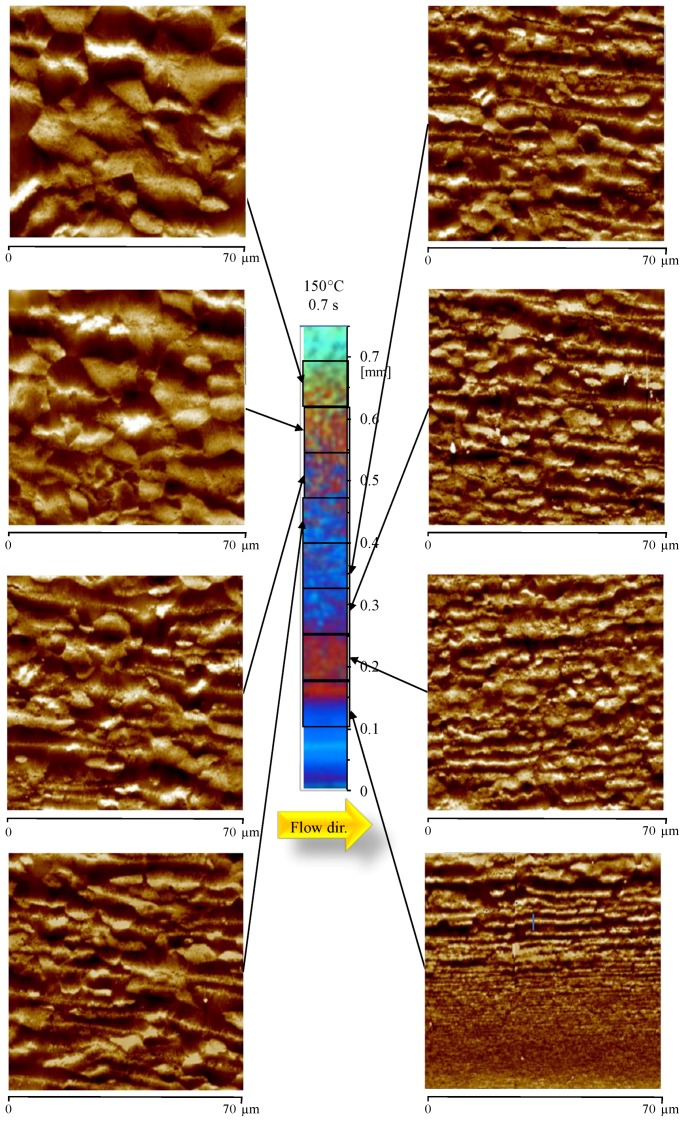
Optical micrograph and AFM height maps, at different distances from the sample surface, of the sample 150-07, at 15 mm downstream the gate.

Figure 6 shows the morphology developed with higher cavity surface temperatures, during the filling (Test 150-07).

The distribution of colored band in the OM image is similar to the one observed for the sample 80-07. The analysis of the AFM maps confirms that, also in this case, structures aligned along the flow direction are already present close to the sample surface, although the sample was obtained with a cooling rate significantly smaller than the Passive case. The layer characterized by thin aligned structures (with thickness smaller than 300 nm) appears to be smaller with respect to the case 80-07. In particular, the AFM map shows that the layer with tightly packed aligned structures ends at 0.15 mm. After this distance, the lateral growth becomes considerable, and the thickness suddenly becomes larger than 300 nm. The layer characterized by a considerable lateral growth is wide, and it ends almost at the same distance (0.50 mm from the sample surface, as shown by AFM map) observed in the case 80-07. 

**Figure 7:**
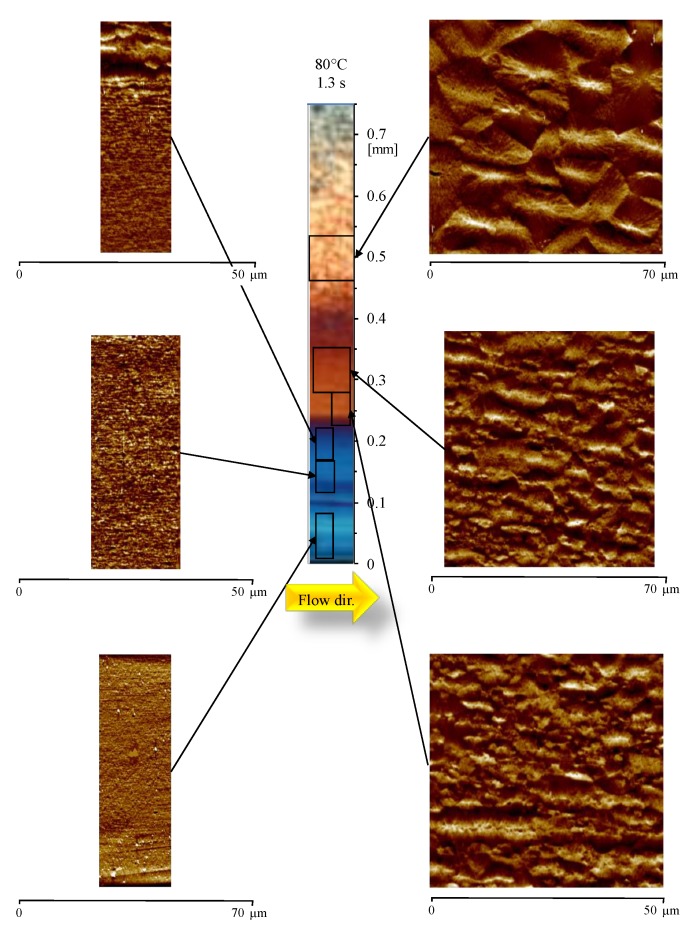
Optical micrograph and AFM height maps, at different distances from the sample surface, of the sample 80-1, at 15 mm downstream the gate.

Figure 7 shows the morphology developed in the sample obtained with 80 °C cavity surface temperature, and 1.3 s heating time. At 0.04 mm from the sample surface, the AFM map shows tightly packed structures aligned along the flow direction. Tightly packed structures, with thicknesses up to 300 nm, are present up to 0.18 mm (i.e. on the border of the OM blue layer). After that, the lateral growth becomes significant (higher than 300 nm thickness), and the structures aligned along the flow direction have a width ranging between 2–4 µm (up to 0.22 mm from the sample surface). Structures aligned along the flow direction can be recognized up to 0.3 mm from the sample surface; after that position, only randomly dispersed spherulites can be recognized.

**Figure 8:**
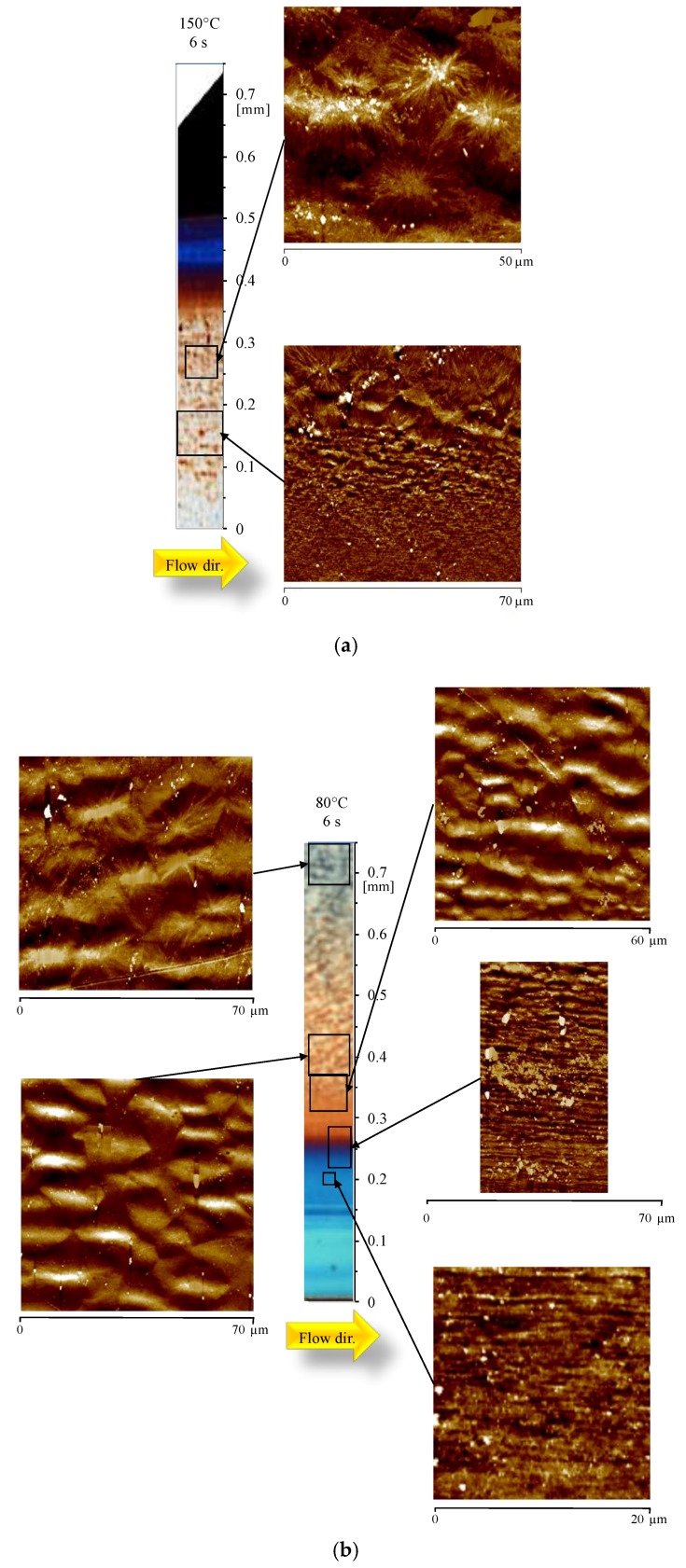
Optical micrograph and AFM height maps, at different distances from the sample surface, of the sample (**a**) 150-6 and (**b**) 80-6 (at 15 mm downstream the gate).

Figure 8a,b shows OM of the samples obtained with 6 s heating time and different cavity surface temperatures, 150 °C and 80 °C respectively. During these tests, the cavity surface temperature was kept high for times comparable with the holding time. In Figure 8a, the AFM map shows that the layer characterized by tightly packed structures (up to 300 nm thickness), aligned along the flow direction, extends up to 0.15 mm, which is the same distance observed for the sample 150-07. The morphology evolves from aligned structures to randomly dispersed spherulites within a short distance. At 0.25 mm from the sample surface, the AFM map only shows randomly dispersed spherulites. The sample 80-6 (Figure 8b) shows a similar distribution: the width of the layer with thin and packed structures extends up to 0.2 mm, the same distance observed for the samples 80-07, and 80-1. The layer in which the lateral growth is significant has a width close to the one observed for the sample 80-1 (see the AFM map at 0.32 mm). After 0.30 mm from the sample surface, only spherulites can be recognized. 

## 4. Discussion

Several studies, in the literature, report morphological results obtained when the polymer chains undergo homogeneous flow field [4,14,29,30,31]. However, few papers discuss the effect of inhomogeneous flow and temperature fields characteristic of the injection molding process [32,33,34]. As mentioned above, the hierarchical structures in polymer products are determined by the flow and temperature fields, and by the macromolecular characteristics. The amount of the stretched/oriented chains induced by the flow in the mold significantly decreases from the sample surface to the sample core. In the sample core, slow cooling and weak flows allow a more complete molecular relaxation of polymer chain, with respect to the layers close to the sample surface. Close to the sample surface, the stronger flow fields favor crystallization in the form of structures aligned along the flow direction, namely shishes, at temperatures significantly higher than in the almost quiescent conditions of the core [4,35,36]. It has been hypothesized that the presence of micellar nuclei, considered precursors for shish formation, is a prerequisite for the prolific formation of shishes [14]; however, the mechanisms that regulate shish formation are still under debate [14,37,38,39]. Keller and coworkers [36] suggest that two critical strain rates determine the final morphology: one below which the polymer chains rest in the coiled conformation, another one above which the polymer chains extend and shish formation occurs. Between these two critical values a significant lateral growth is allowed. Mackley et al [40] ascribed the formation of shishes to the extension of polymer chains, due to high flow intensity; with less intense flow, a significant lateral growth was found. 

Recent studies relate the formation of shishes to the stretched chain network, instead than the extension of a single polymer chain [15,41]. Hsiao and co-workers [4,29] hypothesized that the polymer chains stretched more than a certain critical value could aggregate to form extended chains, the shishes, and the remaining coil polymer chains could then crystallize on the shishes in a folded periodic manner, forming the shish–kebab morphology. Kornfield et al [14] hypothesized that the formation of shishes takes place in two steps: the initiation, and the propagation. During the first step, the high shear stress induces the formation of precursors/nuclei, whose concentration increases with the flow intensity. Thanks to the flow, the long chains are transported on the precursors and attached to them. After that, the flow induces the extension of long chains, that achieve high orientation levels. During the propagation, the attachment of long chains, one upon the other, allows for the growth and formation of the so-called shish [14]. Mykhaylyk et al [42] supposed that the flow promotes the alignment and the aggregation of precursor/nuclei along the flow direction in the form of shishes.

**Figure 9:**
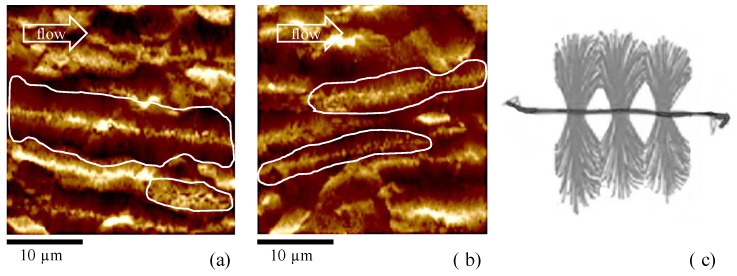
AFM height maps of the samples (**a**) 80-07; and (**b**) 150-07, at 0.50 mm from the sample surface; (**c**) Sketch of cylindritic structure adapted from An et al [34].

All of these theories associate flow intensity with the density of precursors/nuclei, and the arrangement of precursors/nuclei in the space. Under strong flow field, the density of the nuclei is so high and the growth rate of these nuclei is so fast that the crystals get compact very quickly [43] and lateral growth is significantly limited due to the impingement. In these cases, the nucleation of α-phase was found to be predominant with respect to the other crystalline phases [32,34], and shishes were found. This kind of crystallization is based on the unidirectional propagation of a growth front [44], and it characterizes the layers close to the surface of the samples analyzed in this work. Obviously, the larger the distance from the sample surface, the more significant the lateral growth, and subsequently the higher the thickness of the shishes. The smaller the flow intensity, the smaller also the density of precursors/nuclei. As the distance from the sample surface increases, the flow intensity and the cooling rate decreases. As a consequence, the density of α-row nuclei decreases, and lateral growth is allowed. In this case, the growing of other crystalline phases, i.e. β-phase [39,45], in the form of cylindrites, was also observed. In particular, the formation of β-phase, with temperatures close to the ones adopted in this work, and with a shear rate of 1 s^−1^, was already observed for the grade of iPP adopted in this work [46]. Figure 9a,b shows structures that appear aligned along the flow direction, and formed by shish-like core structures with secondary growth of lamellae from the shish-like core in the transverse direction, as also reported in the sketch of Figure 9c. Thus, one can conclude that the structures shown in Figure 9a,b, found in the samples 80-07 and 150-07, can be attributed to the growth of β-phase in the cylindritic form [34,47]. 

The cylindrites are characterized by smaller length than the fibers, as also shown in the figure, since the impingement takes place in both directions with respect to the flow front, probably due to weak flow. The impingement along the flow direction is pointed out in the figure by white boundaries. Also, the cylindrites appear to be less oriented along the flow direction with respect to the structures observed in the layers close to the sample surface. It is very interesting that properly changing the injection molding operating conditions makes it possible to detect β-cylindrites, since, generally, their presence is induced by β-nucleating agent in order to improve the toughness of the sample [48,49].

The AFM maps obtained for the samples produced in this work were analyzed taking into account all of the mentioned theories on the formation of different kinds of morphologies. In particular, on the basis of the height profiles, six areas were identified:

- the area with poorly formed structures close to the sample surface;
- the area with tightly packed structures, shishes with thickness up to 300 ± 50 nm;
- the area with structures aligned along the flow direction, which includes structures presenting a thickness larger than 300 nm and up to about 2 µm, namely shish-kebab;
- the area characterized by cylindritic structures (up to 6 µm thickness);
- the area characterized by randomly dispersed spherulites;
- the overlapping zone, in both the shishes and cylindrites can be also observed.

**Figure 10:**
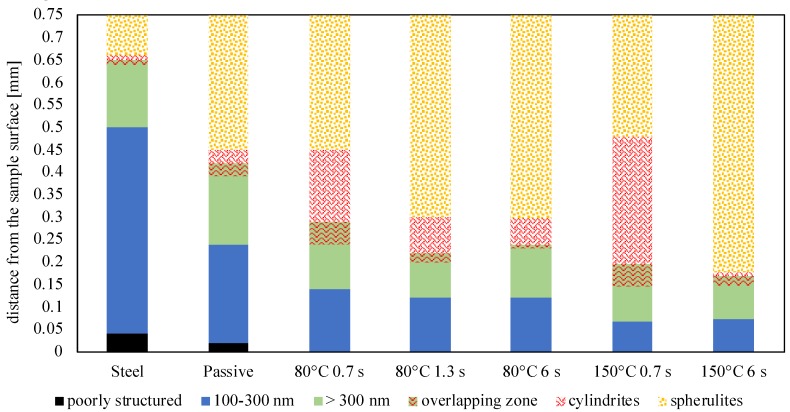
Representation of the layers characterized by the presence of: poorly structured (black), tightly packed shishes (thickness up to 300 ± 50 nm) (blue), less packed shishes (green), cylindrites (red) and spherulites (yellow) as evaluated from AFM acquisitions. There is also an overlapping zone in which different kinds of oriented structures can be detected.

Figure 10 shows the results.

In Figure 10, the sample (named Steel) obtained by replacing the heating device with a steel layer of the same thickness—in other words, the sample obtained in conventional injection molding conditions—is also reported. The Steel sample shows the widest layer characterized by tightly packed shishes, up to 0.5 mm from the sample surface. The layer characterized by thicker aligned structures extends up to 0.66 mm. The width of layers characterized by shishes mainly depends on the fast cooling (the fastest one in this work) that the polymer experienced already during the filling. The Passive sample shows a wide layer with tightly packed shishes (it ends at about 0.25 mm from the sample surface). Such a layer becomes 50% and 70% thinner as the cavity surface temperature T_cs_ increases up to 80°C and 150°C, respectively. The effect of the heating time on the width of this layer is negligible. Again, Passive sample shows the widest layer with less packed shishes (green), such a layer becomes thinner as the T_cs_ increases, although the thickness of such a layer is less sensitive to the cavity surface temperature than the layer with tightly packed shishes (300 nm thickness). 

At a certain distance from the sample surface, which in the case of the Passive sample corresponds with 0.42 mm, cylindritic structures were also found. These structures were found in all the analyzed samples; however, a larger amount was found in the samples obtained with 0.7 s heating time. Generally, the layer characterized by cylindrites is the most sensitive one to the heating time: the longer is the heating time, the smaller is the width of the cylindritic layer. For instance, with T_cs_ = 150 °C and 6 s heating time, this layer becomes very thin (almost disappearing). The β-phase, characteristic of cylindritic structures is a metastable, and thus small changes in the flow and temperature fields would induce transition into α-phase. In particular, an increase of the heating time promotes the transition into spherulites.

**Figure 11:**
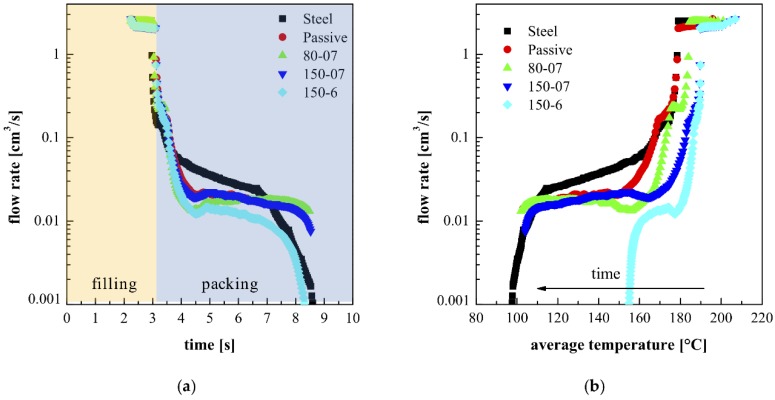
Flow rate vs time (**a**), and average cross section temperature (**b**), for the test Steel, Passive, 80-07, 150-07 and 150-6.

Figure 11a shows the flow rate at the cavity entrance, evaluated by the software code for the injection molding process developed at the University of Salerno, during both the filling and the packing stages. Figure 11b shows the flow rate, namely the integral over the cross section of the velocity profile, plotted versus the average cross section temperature, for the tests Steel, Passive, 80-07, 150-07, and 150-6. Obviously, the polymer experienced the highest temperature at the beginning of the process. For each test, the flow rate was high and essentially constant during the filling stage (namely when temperature is close to the injection temperature). Soon after the cavity filling, the packing stage took place. During such a stage, additional feeding of melt compensated for the shrinkage. In the first tenths of a second of the packing stage, the flow rate decreased from the high values of the filling to much smaller values. 

The boundary condition modification on the cavity surface induces a shift of the flow rates, during each stage of the injection molding process, toward high temperatures. The tests Steel and Passive showed the same flow rate, in the same temperature range, during the filling, and the early stage of the packing. During the packing, the Steel test shows a significantly higher flow rate than the Passive test, since this last test shows a slow heat exchange due to the presence of the heating device acting as an insulator. The tests 80-07 and 150-07 show flow rates similar to the one shown by the Passive test; however, they occur within higher temperatures, depending on the adopted temperature T_cs_. The test 150-6 shows the same flow rate of the test 150-07 during the filling, whereas the packing flow rate decreased monotonically to lower values with respect the other one, as the polymer average temperature in the cross section becomes uniform, and close to the cavity surface temperature (150 °C). For a given initial temperature, the higher the polymer average temperature, the smaller the amount of material needed to compensate for the volume reduction. Thus, the flow rate during the packing, for the sample 150-6, resulted in being smaller with respect to the flow rate of the other tests. 

On the bases of AFM investigations and the calculation of flow rates, it can be hypothesized that the high flow rates during the filling stage, and also during the early stage of packing, are able to induce high molecular orientation. In this case, the formation of the tightly packed shishes is promoted. During the packing stage, the flow rate, certainly smaller than the flow rate during the filling, is able to orient the molecules because the temperatures are smaller. In the inner part of the sample, the intensity of the flow, being smaller with respect to the layers close to the sample surface, allowed a certain lateral growth, and the morphology evolved toward shish-kebab. The thickness of the shish-kebab depends on the impingement of the growing structures. 

For the Steel test, the high flow rate during the packing stage was responsible for the widest extension of the layer characterized by tightly packed shishes. When 0.7 s was adopted as the heating time, the cooling rate was smaller with respect to the Passive case: during the packing step, the same flow rates are attained at a temperature about 20 °C larger than the Passive case (see Figure 11). This fact allows a significant lateral growth, and also the formation of cylindritic structures whose thickness resulted in becoming intermediate between the thickness of the shish-kebab and the mean diameter of the spherulites. With longer heating times, the cooling rate was additionally decreased, and thus, the molecular orientation was expected to be smaller due to the smaller relaxation times at high temperatures. As a result, the layer characterized by cylindrites was thinner, and spherulitical growth was allowed. 

## 5. Conclusions

In this paper, a deep analysis of the morphology developed along the thickness of the samples obtained by injection molding was performed. The samples were obtained adopting different temperatures and times for the cavity surface heating, thus the polymer underwent different thermo-mechanical histories that influence the morphology developed along the sample thickness. Several morphological structures were detected. A poorly structured layer, a layer characterized by tightly packed structures, with a mean thickness smaller than 300 nm, aligned along the flow direction; a layer characterized by less packed aligned structures; a layer characterized by cylindritic structures; and a spherulitical layer, from the sample surface to the core. There is also an area in which both shish and cylindrites could be observed. The formation of these layers was determined by both the cooling rate and the flow field experienced by the polymer during the three main stages of the process, namely the filling, the packing, and the cooling. If the flow field was strong, but the cooling rate is very high, the sudden solidification of the melt occurred, and the polymer chains hadn’t enough time for hierarchical structuring. As a consequence, the poorly structured layer, characteristic of the zones close to the sample surface, formed. If the cooling rate was not fast enough to induce sudden solidification of the polymer, the polymer chains had enough time for structuring. In this case, under a strong flow field, characteristic of the filling stage and the early stage of the packing, tightly packed structures form, namely shishes, with a mean thickness ranging between 100-300 nm. The formation of such packed structures is mainly due to the high density of precursors/nuclei, due to the strong shearing flow. The growth rate of these nuclei is so fast that the crystals become compact very quickly, and the lateral growth is essentially prevented. The larger is the distance from the sample surface, the lower is the flow intensity, and consequently the density of precursor/nuclei. Thus, as the distance from the sample surface increases, the lateral growth becomes significant, and shish-kebab morphology can be observed. The thickness of the layer with packed shishes also depends on the modulation of the cavity surface temperature. The higher the cavity surface temperature, the thinner the layer with packed shishes. In the presence of slow cooling and weak flow, the growth of β-phase is allowed. In these conditions, cylindritic structures appear to be formed by shish-like core structures aligned along the flow direction, with secondary growth of lamellae from the shish-like core in the transverse direction. Generally, the formation of such structures is induced by nucleating agents in order to achieve high toughness. It is very interesting that, in the cases proposed in this work, the formation of such structures was achieved by a proper change in cavity surface temperature. The width of the layer characterized by cylindritic structures was affected by the adopted heating time. The longer the heating time, the thinner the layer with cylindritic structures, and spherulites can be observed for the most of sample thickness. This is because polymer chains had enough time to relax and lose orientation.

## Figures and Tables

**Table 1:** Operating conditions of the injection molding tests. (P = electrical power density; T_cs_ = temperature measured on the cavity surface 6 s after the melt entrance into the cavity; the activation time, *t_a_*, and the heating time, *t_h_*, represent the time during which the heating device was kept active before the first contact of the melt with the cavity surface, and the time during which the heating device was active after the contact of melt with the cavity surface, respectively).

Test Name	P (W/cm^2^)	T_cs_ (°C)	t_h_ (s)	t_a_ (s)
Steel	0	25	0	0
Passive	0	25	0	0
150-07	9.5	150	0.7	2
150-6	9.5	150	6	2
80-07	4	80	0.7	2
80-1	4	80	1.3	2
80-6	4	80	6	2

## Supplementary Materials

The following are available online at http://www.mdpi.com/1996-1944/12/3/424/s1, Figure S1: Temperature evolutions measured by means of a thermocouple type T located on the cavity surface at 20 mm from the cavity entrance during the tests 80-07, 80-1 and 80-6.
