# Correction: Speranza, V. et al. Hierarchical Structure of iPP During Injection Molding Process with Fast Mold Temperature Evolution. *Materials* 2019, *12*, 424

**DOI:** 10.3390/ma13061277

**Published:** 2020-03-11

**Authors:** Vito Speranza, Sara Liparoti, Roberto Pantani, Giuseppe Titomanlio

**Affiliations:** Department of Industrial Engineering, University of Salerno–via Giovanni Paolo II, 132, 84084 Fisciano (SA), Italy; vsperanza@unisa.it (V.S.); rpantani@unisa.it (R.P.); gtitomanlio@unisa.it (G.T.)

The correction reported in the following has to be applied to the paper [1].

Due to an unexpected result of the conversion procedure, the old Figure 8a:


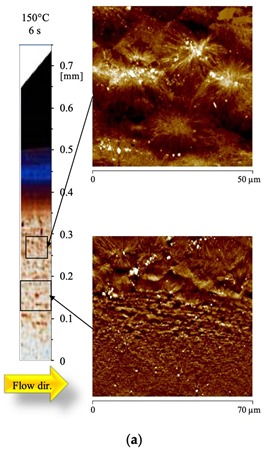


needs to be replaced with the following:


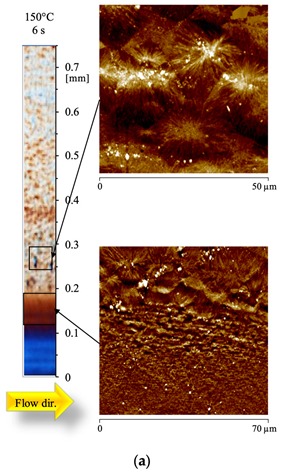


This change does not affect the results and conclusions of the paper. The authors would like to apologize for any inconvenience caused to the readers. 
