# Supplementary material for: Hierarchical Structure of iPP During Injection Molding Process with Fast Mold Temperature Evolution
Source: Materials (Basel). 2019 Jan 30;12(3):424. doi: 10.3390/ma12030424 (PMC6384804; doi:10.3390/ma12030424)
Supplement: Supplementary file 1 [file materials-12-00424-s001.pdf]

materials

Supplementary Materials

Hierarchical structure of iPP during injection molding process with fast mold temperature evolution

Vito Speranza, Sara Liparoti*, Roberto Pantani and Giuseppe Titomanlio

Department of Industrial Engineering, University of Salerno – via Giovanni Paolo II, 132, 84084 Fisciano (SA) Italy; vsperanza@unisa.it; sliparoti@unisa.it; rpantani@unisa.it; gtitomanlio@unisa.it

* Correspondence: sliparoti@unisa.it; Tel.: +39-089-964007

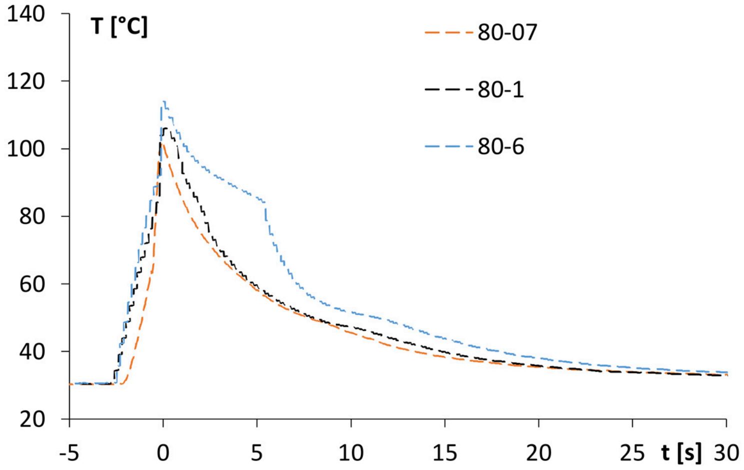

Figure S1. Temperature evolutions measured by means of a thermocouple type T located on the cavity surface at 20 mm from the cavity entrance during the tests 80-07, 80-1 and 80-6.

© 2019 by the authors. Licensee MDPI, Basel, Switzerland. This article is an open access article distributed under the terms and conditions of the Creative Commons Attribution (CC BY) license (http://creativecommons.org/licenses/by/4.0/).
